# Association between visceral adiposity index and kidney stones in American adults: A cross-sectional analysis of NHANES 2007–2018

**DOI:** 10.3389/fnut.2022.994669

**Published:** 2022-09-26

**Authors:** Jiahao Wang, Zhenzhen Yang, Yunjin Bai, Shan Yin, Jianwei Cui, Yunfei Xiao, Jia Wang

**Affiliations:** ^1^Department of Urology, Institute of Urology, West China Hospital, Sichuan University, Chengdu, China; ^2^Department of Clinical Laboratory, Nanchong Central Hospital, Nanchong, China; ^3^Department of Clinical Medicine, North Sichuan Medical College, Nanchong, China

**Keywords:** VAI, NHANES, kidney stones, association, cross-sectional analysis

## Abstract

**Objective:**

To explore the association between Visceral Adiposity Index (VAI) and kidney stones in an American adult population.

**Materials and methods:**

National Health and Nutrition Examination Survey (NHANES) datasets from 2007 to 2018 were used. Participants aged ≥20 years who reported kidney stone history and VAI were included. Weighted proportions, multivariable analysis, generalized additive model (GAM), and spline smoothing were used to evaluate the associations between VAI and kidney stones by adjusting gender, age, race, education, marital status, poverty income ratio, smoking, alcohol, high blood pressure, diabetes, congestive heart failure, cancer, vigorous activity, moderate activity, HEI2015 total score, and energy.

**Results:**

Totally 13,871 American adults were included. All the participants were divided by the VAI into four groups according to the quartile: Q1 (11.96–42.89), Q2 (42.90–74.45), Q3 (74.45–131.43), and Q4 (131.45–611.34). The mean ± standard deviation of the VAI in the four groups were Q1 (29.07 ± 8.22), Q2 (57.53 ± 8.81), Q3 (99.52 ± 16.25), and Q4 (225.92 ± 95.83). In the fully adjusted multivariable model, VAI was positively correlated with urolithiasis [odds ratio (OR) = 1.001; 95% confidence interval (CI) 1.000–1.001]. Compared with the first quartile of VAI, the population in the fourth quartile of VAI had a higher prevalence of kidney stones (OR = 1.329; 95% CI 1.104–1.600). Subgroup analysis detected no significant interaction effect after adjusting for covariates.

**Conclusion:**

The value of VAI is positively correlated with the prevalence of kidney stones, which suggest VAI can be used to assess the potential risk of the prevalence of kidney stones.

## Introduction

Urinary calculi are relatively common in urinary system–related diseases. The incidence of urinary calculi is about 1–20% and increases year by year (1). Survey data from eight countries show that the annual incidence of kidney stones is about 114/100,000–720/100,000, and the prevalence is about 1.7–14.8% (2). Unfortunately, the causes for the high incidence and recurrence rates of urinary calculi are not clear yet, which indirectly leads to difficulties in the prevention and treatment of urinary calculi. The kidney as an important organ in the body retains or excretes most of the body’s metabolites through the filtration and reabsorption function of renal tubular epithelial cells. Disorders of metabolic balance in the body and damages to renal tubular epithelial cells caused by local accumulation of metabolites may be important causes for the formation of urinary calculi (3).

With the changes in dietary structure and daily lifestyle, obesity has gradually become a major problem that plagues human health. Obesity not only brings about body fat accumulation and affects body appearance but also can cause visceral fat accumulation and affect the functions of the corresponding organs. Obesity is related to most of the chronic diseases in the body and is a risk factor for hyperuricemia (4). In addition to relevant cardiovascular diseases caused by obesity, excessive body fat accumulation may increase uric acid production in the serum and inhibit its excretion, eventually leading to uric acid metabolism disorder and affecting kidney function (5). The obesity-caused changes in body mass index (BMI) and waist circumference are positively associated with the risk of kidney stone hospitalization, and the enlarged waist circumference is an independent risk factor for increased risk of kidney stone hospitalization (2).

Visceral Adiposity Index (VAI), a new index used to assess the visceral fat level, is used clinically to assess organ fat. VAI evaluates visceral fat levels by combining waist circumference, BMI, triglycerides, and high-density lipoprotein (6). VAI is closely related to hyperuricemia regardless of the type of metabolic obesity (5). In patients with type 2 diabetes, VAI is also connected with urinary albumin. Compared with the triglyceride/high-density lipoprotein-cholesterol (TG/HDL-c) ratio, VAI has a similar predictive power for the risk of albuminuria (7). VAI is a fast and reliable indicator for evaluating early kidney injury in patients with type 2 diabetes (8). Studies have reported that VAI is also strongly associated with CKD and BPH (9, 10).

The calculation of VAI includes TG, WC, HDL, and BMI. These components are closely related to metabolism, and these components or metabolism are closely related to kidney stones (2, 11). As a comprehensive index containing these components, VAI is closely related to metabolism. Nevertheless, there is limited evidence about the relationship between VAI and kidney stones. Therefore, we investigated the association between VAI and nephrolithiasis by analyzing National Health and Nutrition Examination Survey (NHANES) cross-sectional data. We hypothesize that lower VAI is associated with a lower prevalence of kidney stones.

## Materials and methods

### Study design and participants

The NHANES conducted by the Centers for Disease Control and Prevention (CDC) is a research project that evaluates the health and nutritional status of Americans participating in the survey through interviews, examinations, and laboratory research. The design, data collection procedures, sample weights, and informed consents are elaborated at the National Center for Health Statistics (NCHS), from which related data are publicly available (12).

We extracted data from the NHANES database at 6 consecutive periods from 2007 to 2018 (the period from 1999 to 2006 that did not include issues such as urinary stones or visceral fat assessment was excluded). We initially selected 59,842 participants and included 34,770 participants over the age of 20 years. First, after pregnant participants (*n* = 372) and participants with incomplete kidney stone questionnaires (*n* = 91) were excluded, there were 34,307 participants left. Then by removing those with empty VAI (*n* = 20,155), 14,152 participants were left. Finally, 13,871 participants were included for analysis after removing outliers (less than 1% and more than 99% of data were treated as outliers). All six consecutive periods of NHANES research projects included were approved by the Research Ethics Review Committee of NCHS.

### Outcome and exposure factor

The main outcome indicator was whether the participant had urinary calculi. By limiting the research time between 2007 and 2018, we screened out the questionnaires related to urinary calculi in the NHANES system that had answer “Yes” to the question “Have you/Has sample person (SP) ever had kidney stones.”

The major exposure factor was the VAI, and its value was used as the primary variable. VAI was calculated based on the sex-specific mathematical model: VAI = $\left(\frac{\text{WC}}{36.58+\left(1.89\times \text{BMI}\right)}\right)\times \left(\frac{\text{TG}}{0.81}\right)\times \left(\frac{1.52}{\text{HDL}}\right)$ for women, and VAI = $\left(\frac{\text{WC}}{39.68+\left(1.88\times \mathrm{BMI}\right)}\right)\times \left(\frac{\text{TG}}{1.03}\right)\times \left(\frac{1.31}{\text{HDL}}\right)$ for men (13). The values of VAI calculated thereby are listed in Table 1. The VAI mainly reflects the visceral fat content of the body. A higher VAI means a larger visceral fat content and predicts a higher incidence of cardiovascular diseases.

**TABLE 1:** Characteristics of participants divided by quartile of VAI: NHANES 2007–2018.*^†^

Characteristics	Q1 (11.96–42.89)	Q2 (42.90–74.45)	Q3 (74.45–131.43)	Q4 (131.45–611.34)
VAI (mean ± SD)	29.07 ± 8.22	57.53 ± 8.81	99.52 ± 16.25	225.92 ± 95.83
Age (years, mean ± SD)	45.13 ± 17.44	48.32 ± 17.07	48.72 ± 16.50	49.81 ± 15.37
20–34 (%)	35.08	25.88	23.23	19.12
35–49 (%)	24.57	27.06	29.25	30.28
50–64 (%)	23.51	27.10	27.52	31.58
≥65 (%)	16.84	19.96	20.00	19.02
**Gender (%)**				
Male	49.43	50.69	47.57	47.26
Female	50.57	49.31	52.43	52.74
**Race (%)**				
Mexican American	5.63	8.17	10.09	10.97
Other Hispanic	4.99	5.98	6.90	6.28
Non-Hispanic white	66.83	66.63	65.20	69.51
Non-Hispanic black	13.24	11.65	9.83	6.22
Other races	9.31	7.56	7.98	7.03
Poverty Income Ratio (mean ± SD)	3.18 ± 1.64	3.06 ± 1.66	2.89 ± 1.62	2.80 ± 1.63
≤1.3 (%)	17.44	19.50	20.51	23.59
>1.3 and ≤3.5 (%)	31.19	31.97	36.75	34.13
>3.5 (%)	44.08	41.73	35.51	35.13
Missing (%)	7.28	6.79	7.23	7.15
Energy (kcal)	2217.36 ± 972.27	2153.39 ± 938.39	2135.70 ± 1003.54	2153.53 ± 974.02
HEI2015_TOTAL_SCORE	52.96 ± 14.23	51.09 ± 13.99	49.23 ± 13.57	48.73 ± 12.84
**Education (%)**				
Less than 9th grade	3.72	5.05	5.95	7.45
9–11th grade	7.98	10.12	11.36	13.39
High school graduate	19.77	22.01	24.35	25.51
Some college	28.36	31.58	32.01	31.02
College graduate or above	40.16	31.24	26.33	22.64
**Marital Status (%)**				
Married	54.44	55.51	55.83	58.54
Widowed	4.28	5.27	5.38	6.80
Divorced	8.26	11.14	11.49	11.40
Separated	1.65	2.47	2.72	2.36
Never married	22.60	17.38	16.35	13.17
Living with partner	8.78	8.24	8.23	7.72
**Smoking (%)**				
<100 cigarettes in life	59.87	57.20	52.48	50.86
≥100 cigarettes in life	40.13	42.80	47.52	49.14
**Alcohol (%)**				
<12 drinks/year	14.45	17.78	17.11	21.22
≥12 drinks/year	58.17	60.37	60.09	58.40
Missing	27.38	21.85	22.79	20.38
**High Blood Pressure (%)**				
No	79.79	68.72	64.15	54.78
Yes	20.21	31.28	35.85	45.22
**Diabetes (%)**				
No	94.76	91.23	86.50	79.39
Yes	3.68	6.57	11.38	17.03
Missing	1.57	2.20	2.12	3.58
**Congestive Heart Failure (%)**				
No	98.55	98.22	97.35	96.36
Yes	1.45	1.78	2.65	3.64
Cancer (%)				
No	90.93	90.43	90.73	88.48
Yes	9.07	9.57	9.27	11.52
**Vigorous activity (%)**				
No	62.54	72.10	79.78	85.45
Yes	37.46	27.90	20.22	14.55
**Moderate activity (%)**				
No	45.92	53.32	55.96	63.08
Yes	54.08	46.68	44.04	36.92
**Kidney Stones (%)**				
No	93.22	90.79	88.71	87.39
Yes	6.78	9.21	11.29	12.61

*Mean ± SD for continuous variables, and *p* value calculated by weighted *t*-test.

^†^% for categorical variables, and *p* value calculated by weighted Chi-square test.

### Covariates

To make the association between kidney stones and VAI robust, we adjusted the following covariates: age, gender, race, marital status, education, poverty income ratio (PIR), smoking, alcohol; vigorous activity, moderate activity, HEI2015 total score, energy, and some self-reported medical conditions (all classified as yes/no). Age was categorized as 20–34, 35–49, 50–64, and >65 years. Races included Mexican American, other Hispanic, non-Hispanic white, non-Hispanic black, and other races. PIR was set at ≤1.3, >1.3 and ≤3.5, >3.5. Smoking was classified as <100 or ≥100 cigarettes in life. Alcohol drinking was defined as <12 or ≥12 alcohol drinks per year. Marital status was divided into married, widowed, divorced, separated, never married, and living with a partner. Education level included less than 9th grade, 9th–11th grade, high school graduate, some college, and college graduate or above. The medical conditions included diabetes, high blood pressure (HBP), congestive heart failure (CHF), and cancer. Dummy variables were used to indicate missing covariate values for variables with more than 2% missing data.

### Statistical analyses

In NHANES, sampling weights are often used to account for more complex study designs. The interview and test weights recommended by the CDC guidelines^1^ were used (12, 14–16). Continuous variables were presented as mean ± standard deviation (SD), while categorical variables were expressed as proportions. Analytical comparisons were performed using a weighted *t*-test and Chi-square test.

The association of VAI with nephrolithiasis was analyzed using logistic regression models with or without adjustment for various potential confounders. Model 1 was not adjusted. Model 2 was adjusted for age, gender, and race. Model 3 was adjusted for gender, age, race, education, marital, PIR, smoking, alcohol, HBP, diabetes, CHF, cancer, HEI2015 total score, energy, vigorous activity, and moderate activity. To better explore the association between VAI and kidney stones, we conducted multivariable logistic regression with VAI as a continuous and categorical variable (divided into quarters). The trends were estimated by treating VAI quartiles as a continuous variable. We treated less than 1% and more than 99% of data as outliers and excluded. Then, we further analyzed whether there was a nonlinear association between VAI and the risk of kidney stones by using generalized additive model (GAM) and curve fitting. If yes, a two-piecewise linear regression model was conducted to calculate the threshold effect of the VAI on kidney stones in terms of the smoothing plot and used a recursive method to automatically calculate the infection point, where the maximum model likelihood was used. Finally, subgroup analyses were performed using hierarchical logistic regression models for all potential confounders listed in the baseline table.

We used the statistical software packages R^2^ (the R Foundation) and EmpowerStats^3^ (X&Y Solutions Inc.) in all analyses. Two-tailed *p* values <0.05 were considered significant.

## Results

### Baseline characteristics

A total of 13,871 American adults were included, and divided into four quartiles according to VAI: Q1 (11.96–42.89), Q2 (42.90–74.45), Q3 (74.45–131.43), and Q4 (131.45–611.34). The mean ± SD of the VAI in the four groups are Q1 (29.07 ± 8.22), Q2 (57.53 ± 8.81), Q3 (99.52 ± 16.25), and Q4 (225.92 ± 95.83). Table 1 shows the baseline characteristics of different VAI groups. According to the VAI categories, kidney stones ever accounted for 6.78, 9.21, 11.29, and 12.61% in groups Q1, Q2, Q3, and Q4, respectively.

### Multivariate regression analysis

The multivariate regression analyses with different adjustments for the effect of confounders on the correlation showed that VAI was positively correlated with kidney stones in model 1 [OR (95%CI) = 1.002 (1.001–1.003)], model 2 [1.002 (1.001–1.002)], and model 3 [1.001 (1.000–1.001)]. Besides, compared to Q1, the participants in group Q4 (131.45–611.34) had a significantly increased risk of developing kidney stones in model 1 [1.879 (1.589–2.222)], model 2 [1.1.644 (1.384–1.953)], and model 3 [1.329 (1.104–1.600)]. *P* for trend in all three models was less than.05 (Table 2).

**TABLE 2:** Association of VAI with kidney stones.

Exposure	Model 1*	Model 2^†^	Model 3^‡^
VAI (continuous)	1.002 (1.001, 1.003) <0.00001	1.002 (1.001, 1.002) <0.00001	1.001 (1.000, 1.001) 0.00818
**Quartile of VAI**			
Q1 (4.20–42.09)	Ref	Ref	Ref
Q2 (42.09–73.63)	1.347 (1.129, 1.608) 0.00095	1.247 (1.042, 1.491) 0.01587	1.173 (0.973, 1.414) 0.09432
Q3 (73.64–130.54)	1.661 (1.400, 1.970) < 0.00001	1.501 (1.262, 1.787) < 0.00001	1.322 (1.101, 1.588) 0.00280
Q4 (130.56–611.34)	1.879 (1.589, 2.222) < 0.00001	1.644 (1.384, 1.953) < 0.00001	1.329 (1.104, 1.600) 0.00263
*P* for trend	< 0.00001	<0.00001	0.00164

*Model 1: not adjusted.

^†^Model 2: adjusted for gender; age; race.

^‡^Model 3: adjusted for gender; age; race; education; marital; PIR; smoking; alcohol; HBP; diabetes; CHF; cancer; vigorous activity; moderate activity, energy, and HEI2015 total score.

### Nonlinear analysis

**FIGURE 1:**
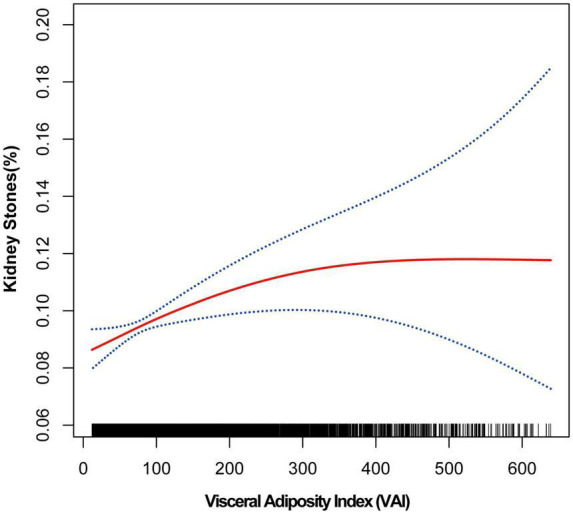
Smooth curve fitting of kidney stones and VAI Smooth curve fitting was performed using GAM to explore the association between kidney stones and VAI.

The association between VAI and kidney stones was investigated by using GAM, smooth curve fitting, and piecewise linear regression (Table 3 and Figure 1). Figure 1 demonstrated the results of the fully adjusted model. We treated less than 1% and more than 99% of data as outliers and excluded. The plotting revealed that VAI and kidney stone incidence were under a curve relationship. With the increase of VAI, the risk of developing nephrolithiasis increased parabolically and leveled off gradually after VAI reached a certain value. Then, we further conducted piecewise linear regression to find the point of infection (Table 3). When VAI was <75.130, each unit increase in VAI increased 5‰ risk of developing kidney stones [1.005 (1.001–1.009)]. When VAI was >75.130, the risk of kidney stones was steady [1.000 (1.000–1.001)]. *P* for likelihood-ratio test was less than 0.05. These results indicate that the association between VAI and kidney stones is nonlinear.

**TABLE 3:** Results of binary logistic regression and piecewise linear regression.*

Outcome: kidney stones	Adjusted OR (95% CI)	*p* value
Fitting by binary logistic regression model	1.001 (1.000, 1.001)	0.0082
**Fitting by piecewise linear regression model**		
Inflection point	75.130	
VAI < 75.130	1.005 (1.001, 1.009)	0.0084
VAI > 75.130	1.000 (1.000, 1.001)	0.3078
Log likelihood ratio test	0.025	

*All models were adjusted for: gender; age; race; education; marital; PIR; smoking; alcohol; HBP; diabetes; CHF; cancer; vigorous activity; moderate activity, energy, and HEI2015 total score.

### Subgroup or interaction analyses

**FIGURE 2:**
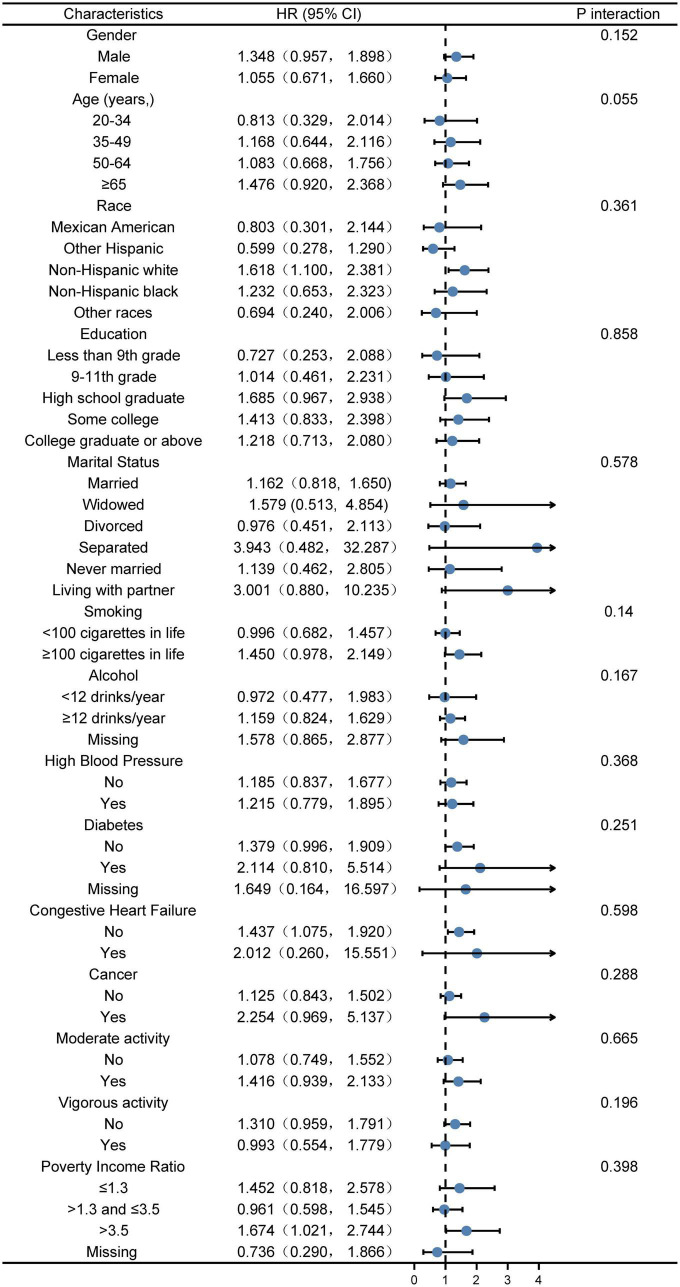
Stratified logistic regression analysis to identify variables that modify the correlation between VAI and kidney stones A subgroup and interaction analyses with VAI as a categorical variable (divided into quarters), for the part of VAI <75.130, compared Q4 with Q1. Adjusted for gender, age, race, poverty income ratio, education, marital status, smoking, alcohol, vigorous activity, moderate activity, diabetes, HBP, CHF, cancer, energy, and HEI2015 total score. The model is not adjusted for the variable itself in each stratification.

According to the piecewise linear regression analysis, when VAI was <75.130, VAI was positively correlated with the risk of kidney stones (*p* = 0.0084) and the relationship was approximately linear. We performed subgroup and interaction analyses with VAI as a categorical variable (divided into quarters), for the part of VAI < 75.130, with model 3. The plotting uncovered that in subgroup analysis, compared Q4 with Q1, no significant interaction effect was detected after adjusting for covariates (Figure 2). Same results were found in models 1 and 2 (Supplementary Figures 1, 2).

## Discussion

We investigated the association between VAI and the risk of developing kidney stones by analyzing large population data in NHANES. Results demonstrate that kidney stones are closely related to VAI, showing a positive correlation. In other words, higher VAI was associated with a higher prevalence of kidney stones. For those with VAI <75.130, each unit increase in VAI is associated with a 5‰ increase in the risk of kidney stones. For those with VAI >75.130, however, increasing VAI shows a trend toward an increased risk of kidney stones, although not significant. Therefore, we present a close relationship between the increase in VAI and the higher risk of kidney stones. VAI may be a practical indicator for clinical assessment of the risk of kidney stones.

As an indicator of the visceral fat level in the body, VAI can more accurately assess the visceral fat content, which is closely related to the health status of most human bodies. Visceral fat accumulation indirectly reflects the abnormality of matter and energy metabolism in the body. Compared with indicators such as BMI and WC, VAI has a stronger predictive ability for metabolic disorders (5). Reportedly, VAI has the potential value of identifying metabolic disorder syndromes (17). VAI even plays an important role in assessing ED, lung function, cardiovascular diseases, diabetes, chronic kidney disease (CKD), and the degree of hepatic steatosis (10, 13, 18–21).

Kidney stones are a multifactorial disease. Reportedly, disturbance of Ca^2+^ level in the body caused by abnormal parathyroid function can exacerbate the formation of kidney stones (22, 23). In addition to the above factors, metabolism plays an important role in the formation of kidney stones. Some scholars believe that diet structure and diet type are inseparable from the formation of kidney stones. Adhering to normal BMI, drinking enough water, eating more fruits and vegetables, eating more low-fat dairy products and adequate calcium intake, avoiding regular sugar-sweetened beverages, and maintaining a healthy lifestyle all can help reduce the incidence of kidney stones rate over 50% (24). Metabolic syndrome, characterized by various metabolic disorders in the body, can increase urinary calcium, uric acid, and oxalate excretion, and reduce urinary citrate excretion, leading to the formation of calcium oxalate and uric acid stones (25–27). Obesity is often a manifestation of abnormal lipid metabolism in the body, and a higher proportion of fat is positively associated with a higher risk of kidney stones in both men and women (28, 29). At the same time, different lipoprotein levels in the body affect different stone types, such as the significant prevalence of uric acid stones caused by high-density lipoprotein and triglyceride levels (11). Animal experiments show that peritubular fat accumulation can increase the accumulation of local pro-inflammatory adipocytokines and macrophages, thereby increasing the formation of kidney stones (30). In addition, the formation of kidney stones was significantly increased in a mouse model after the lipid metabolism-related protein fatty acid binding protein 4 (FABP4) was knocked out (31).

Due to the high prevalence and recurrence rates, kidney stones have brought enormous economic pressure to the health prevention and treatment system. Problems such as renal colic and renal function damage that accompany kidney stones have greatly challenged human health. Therefore, the prevention of kidney stones is an indispensable part of the current health prevention and treatment system. This study is based on a large population-based analysis of the association between VAI and kidney stone prevalence. Results show a certain relationship between VAI and the prevalence of kidney stones, which theoretically supports the use of VAI to assess the potential risk of developing kidney stones in the body. However, the study still has certain limitations. First, due to the cross-sectional study design, we cannot draw a causal relationship between VAI and kidney stone prevalence. Furthermore, despite the adjustment for some potential confounders, we still cannot completely rule out the confounding caused by some unknown variables.

## Conclusion

Visceral Adiposity Index is inversely associated with the prevalence of kidney stones, suggesting that maintaining a lower VAI is related to a lower risk of kidney stones. VAI may be a practical indicator for clinical assessment of the risk of kidney stones. Nevertheless, more high-quality prospective studies are needed to clarify the underlying mechanism between VAI and nephrolithiasis.

## Data availability statement

The original contributions presented in this study are included in the article/Supplementary material, further inquiries can be directed to the corresponding author.

## Ethics statement

The studies involving human participants were reviewed and approved by the National Center for Health Statistics (NCHS) Research Ethics Review Committee. The patients/participants provided their written informed consent to participate in this study.

## Author contributions

JW and JHW: conceptualization and methodology. SY, JHW, and ZZY: data acquisition. JHW, SY, YJB, ZZY, JWC, and YX: software and formal analysis. JHW and SY: writing—original draft. JW: data curation and supervision. All authors: writing—review and editing and read and approve the final manuscript.

## Footnotes

1
https://wwwn.cdc.gov/nchs/nhanes/tutorials/default.aspx

2
http://www.R-project.org

3
http://www.empowerstats.com

## Supplementary material

Supplementary Figure 1 Stratified logistic regression analysis to identify variables that modify the correlation between VAI and kidney stones. A subgroup and interaction analyses with VAI as a categorical variable (divided into quarters), for the part of VAI <75.130, compared Q4 with Q1. Not adjusted.

Supplementary Figure 2 Stratified logistic regression analysis to identify variables that modify the correlation between VAI and kidney stones. A subgroup and interaction analyses with VAI as a categorical variable (divided into quarters), for the part of VAI <75.130, compared Q4 with Q1. Adjusted for gender; age and race. The model is not adjusted for the variable itself in each stratification.
