# Supplementary figures and images for: Association between visceral adiposity index and kidney stones in American adults: A cross-sectional analysis of NHANES 2007–2018
Source: Front Nutr. 2022 Sep 26;9:994669. doi: 10.3389/fnut.2022.994669 (PMC9548983; doi:10.3389/fnut.2022.994669)

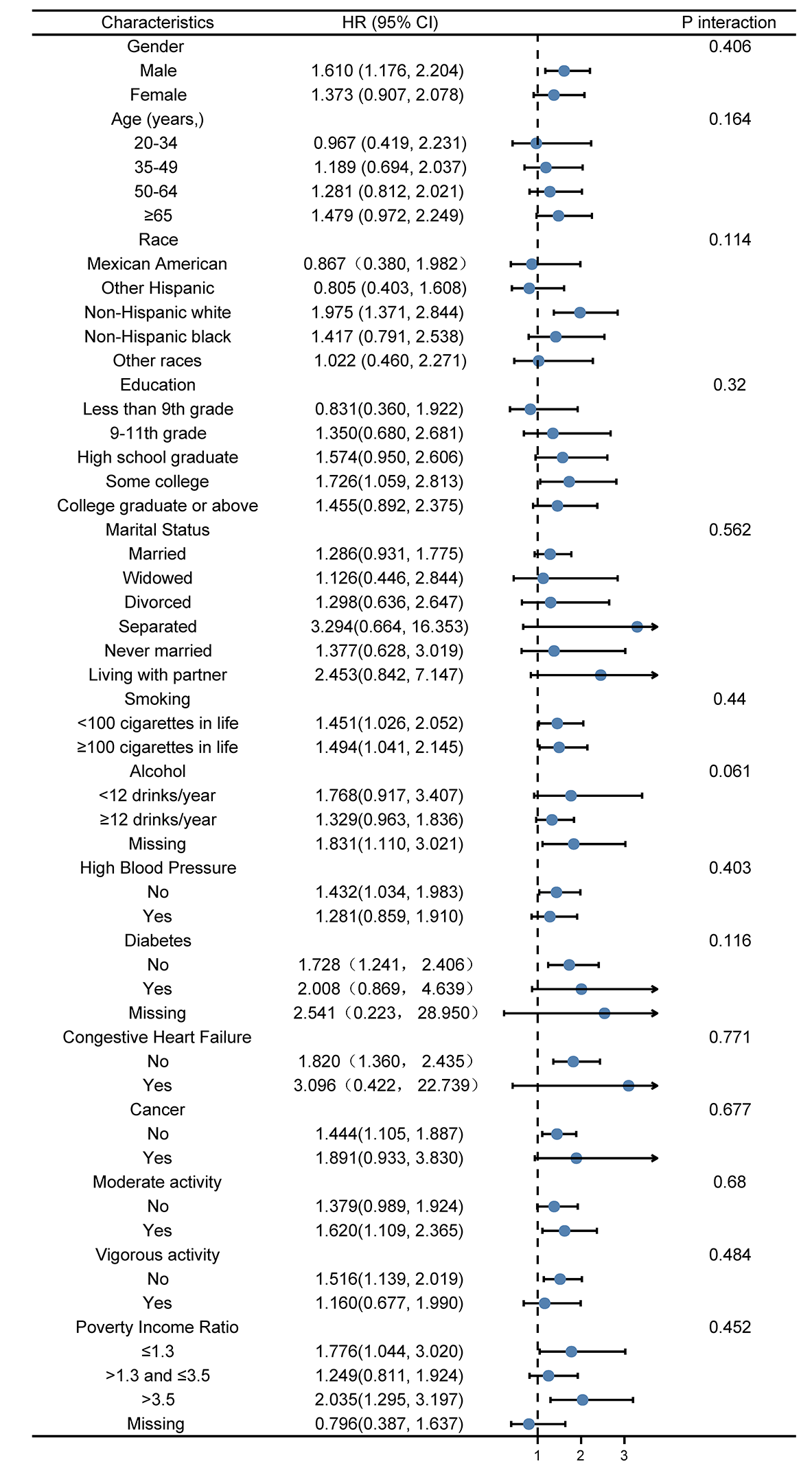

Supplement: Supplementary Figure 1 — Stratified logistic regression analysis to identify variables that modify the correlation between VAI and kidney stones. A subgroup and interaction analyses with VAI as a categorical variable (divided into quarters), for the part of VAI <75.130, compared Q4 with Q1. Not adjusted. [file Image_1.TIFF]

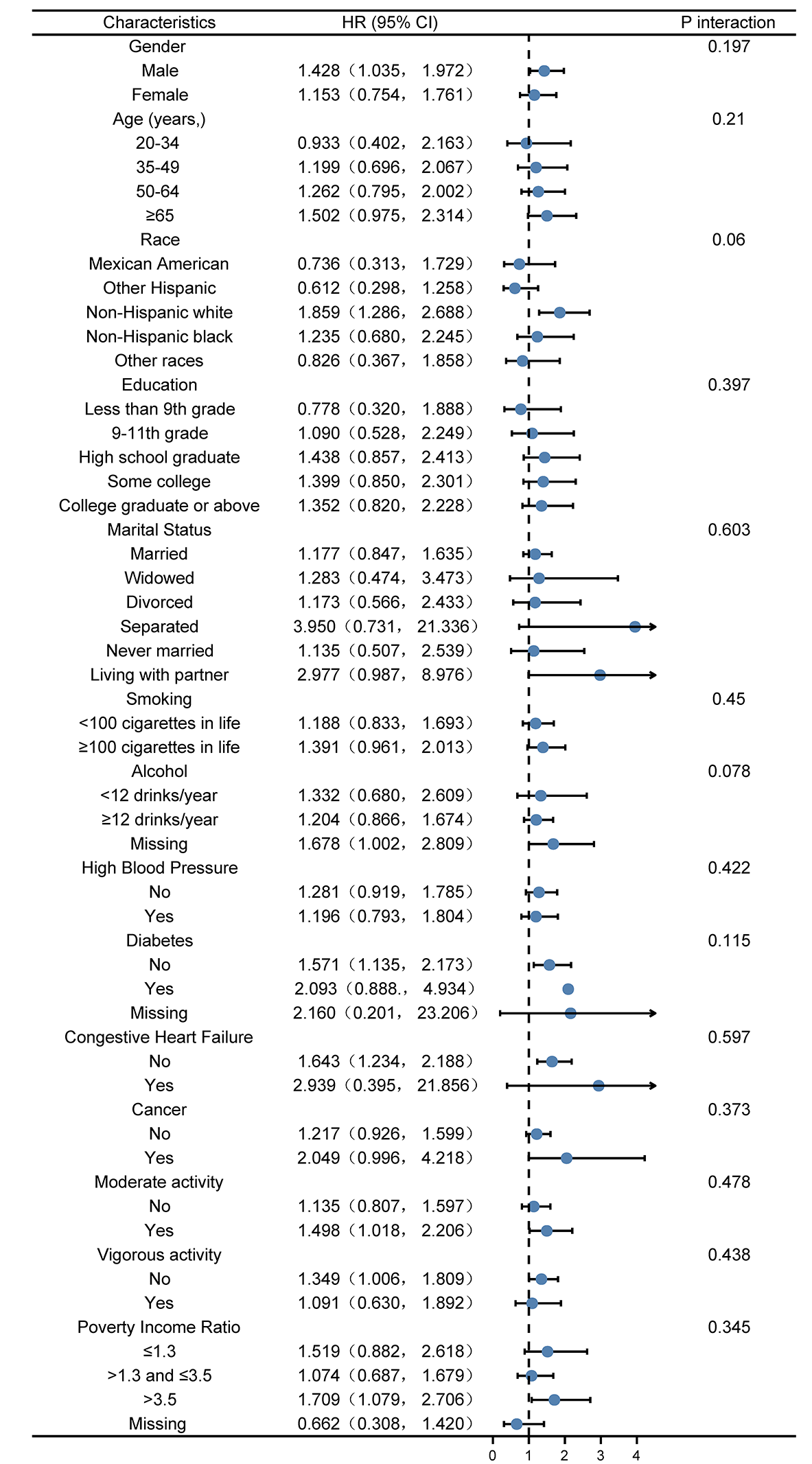

Supplement: Supplementary Figure 2 — Stratified logistic regression analysis to identify variables that modify the correlation between VAI and kidney stones. A subgroup and interaction analyses with VAI as a categorical variable (divided into quarters), for the part of VAI <75.130, compared Q4 with Q1. Adjusted for gender; age and race. The model is not adjusted for the variable itself in each stratification. [file Image_2.TIFF]
